# Impact of Polypyrrole Functionalization on the Anodic Performance of Boron Nitride Nanosheets: Insights From First-Principles Calculations

**DOI:** 10.3389/fchem.2021.670833

**Published:** 2021-04-28

**Authors:** Chidera C. Nnadiekwe, Ismail Abdulazeez, Muhammad Haroon, Qing Peng, Almaz Jalilov, Abdulaziz Al-Saadi

**Affiliations:** ^1^Chemistry Department, King Fahd University of Petroleum and Minerals, Dhahran, Saudi Arabia; ^2^Physics Department, King Fahd University of Petroleum and Minerals, Dhahran, Saudi Arabia; ^3^K.A CARE Energy Research & Innovations Center at Dhahran, Dhahran, Saudi Arabia

**Keywords:** metal-ion batteries, boron nitride nanosheets, DFT, polypyrrole, cell voltage, energy storage capacity

## Abstract

Lithium-ion batteries (LIBs) have displayed superior performance compared to other types of rechargeable batteries. However, the depleting lithium mineral reserve might be the most discouraging setback for the LIBs technological advancements. Alternative materials are thus desirable to salvage these limitations. Herein, we have investigated using first-principles DFT simulations the role of polypyrrole, PP functionalization in improving the anodic performance of boron nitride nanosheet, BNNS-based lithium-ion batteries and extended the same to sodium, beryllium, and magnesium ion batteries. The HOMO-LUMO energy states were stabilized by the PP functional unit, resulting in a significantly reduced energy gap of the BNNS by 45%, improved electronic properties, and cell reaction kinetics. The cell voltage, ΔE_cell_ was predicted to improve upon functionalization with PP, especially for Li-ion (from 1.55 to 2.06 V) and Na-ion (from 1.03 to 1.37 V), the trend of which revealed the influence of the size and the charge on the metal ions in promoting the energy efficiency of the batteries. The present study provides an insight into the role of conducting polymers in improving the energy efficiency of metal-ion batteries and could pave the way for the effective design of highly efficient energy storage materials.

## Introduction

Rechargeable batteries are green energy sources that have significantly influenced the civilization of this modern-day world (Dunn et al., 2011; Yang et al., 2011; Larcher and Tarascon, 2015; Grey and Tarascon, 2017). One of the efficient rechargeable batteries is the lithium-ion battery which has wide applications in portable electronic devices due to its high energy density, smaller volume, low self-discharging rate, high operating voltage, absence of memory effect, and long cycle life (Armand and Tarascon, 2008). Lithium-ion batteries have displayed superior performance over other types of rechargeable batteries (Prosini et al., 2015; Johannes et al., 2016; Kino et al., 2016). Despite the high advances of lithium-ion batteries, several undesired limitations and difficulties abound therewith to weaken the further advancements of lithium-ion battery technology. Such limitations and difficulties include a limited lifetime, poor performance at low temperatures, and the rapidly depleting lithium mineral reserves which is the most daring drawback of Li-ion technology. The large-scale application of Li-Ion batteries has resulted in a high cost of Lithium minerals and may represent the dead-end for the Li-ion batteries technology. For instance, about $\frac{1}{4}$ of the world's lithium precursor materials are consumed to manufacture Li-ion batteries, birthing the sharp hike in the cost of lithium carbonate (Naumov and Naumova, 2010; Grosjean et al., 2012; Martin et al., 2017). Therefore, extensive researches have been devoted to alternative elements for ion-batteries technology (Barker et al., 2003; Levi et al., 2009; Er et al., 2014; Hosseinian et al., 2017). Due to low cost, non-toxicity, and almost unlimited Na mineral reserves, the Sodium-ion batteries (SIBs) are expected to replace the Lithium-ion batteries (LIBs) (Barker et al., 2003; Levi et al., 2009; Palomares et al., 2012; Pan et al., 2013; Er et al., 2014; Yan et al., 2019). However, some of the drawbacks of the SIBs include a low capacity of electrical energy storage, rates of charging and discharging, and low energy density (Landi et al., 2009).

The discovery of nanotechnology and nanomaterials over the last decade has immensely accelerated the process and development of ion-batteries by offering high-performance electrodes (Ahmadi Peyghan et al., 2013; Peyghan et al., 2013; Shao et al., 2015; Jeong et al., 2016; Jiang et al., 2016). Nanomaterials such as graphene, fullerenes, nanocones, nanoeggs, graphenylene, and nanotubes, have been investigated for application as nano-electrodes (Peyghan and Noei, 2014; Chen et al., 2016; Gurung et al., 2016). According to Lee et al. (2010), carbon nanotubes have shown superior lithium capacities and energy storage more than graphite. However, the performance of electrode nanomaterials has been shown to improve through structural modifications such as chemical functionalization, and/or doping with foreign materials (Wu et al., 2011; Qie et al., 2012; Liu et al., 2013). For instance, Qie et al. (2012) and Hardikar et al. (2014) reported that the performance of a Li-ion battery was significantly improved by replacing the carbon atoms of graphene with boron or nitrogen atoms. Furthermore, Yu (2013) established that the nitrogen-substituted graphene having double vacancies can improve significantly the Li-ion battery performance. Chen et al. (2017) experimentally investigated the role of nitrogen-enrichment of hard carbon for application as a durable anodic material for high-performing potassium-ion batteries (Chen et al., 2017). A high capacity rate of 154 mAhg^−1^ at 72 cycles and a prolonged cycle life of 4 × 10^3^ cycles were reported showing efficient capability rate and longest cycle life for potassium-ion batteries. Similarly, Xu et al. (2016) facilely fabricated hierarchical N/S-codoped microspheres of carbon from cellulose/polyaniline composite through a green and cheap approach for high-performance SIBs. The material showed an enhanced conductivity and expanded interlayer distance with a reversible capacity of as high as 280 mAhg^−1^ at 30 mAhg^−1^, and an excellent 130 mAhg^−1^ performance at 10 Ag^−1^. Generally, less dense elements such as nitrogen and boron can produce appropriate sites of adsorption of ions that exhibit weak interaction with the bare graphene. Aside from nanomaterials doped with boron and nitrogen, boron nitride (BN) nanostructures such as BN nanotubes, BN nanocages, and BN nanosheets have extensively been exploited for ion-battery electrodes application (Shi et al., 2007; Ouyang et al., 2009; Beheshtian et al., 2013; Peyghan et al., 2013).

Boron Nitride nanostructures have shown appealing mechanical, lower toxicity, wide bandgap, high thermal conductivity, high oxidation resistance constant, and excellent chemical stability (Chen et al., 2009; Golberg et al., 2010; Hosseinian et al., 2017). The BN nanostructures have attracted significant attention from researchers owing to their properties and their applications spans across chemical sensors, hydrogen storage, and as an anode material for lithium-ion batteries as proposed by Hosseini et al. (2017), Beheshtian et al. (2011), and Shokuhi Rad and Ayub (2016). Specifically, hexagonal BN nanosheet also known as white graphene is an isostructural graphene arrangement produced through the chemical vapor deposition technique (Kang Kim et al., 2011). The 2D hexagonal BN monolayer has been extensively investigated including the electronic structure properties (Peng and De, 2012), mechanical properties (Peng et al., 2012a,b, 2013a), phase change (Peng, 2018), and radiation hardness (Peng et al., 2013b). The BN nanosheet has shown superior all-encompassing properties over graphene such as biocompatibility (Ciofani et al., 2012), thermal and chemical stability (Zeng et al., 2010), and increased carrier mobility (Sławińska et al., 2010). The potential application of BN nanosheets (BNNS) as ion-batteries anode materials have widely been proposed and reported by many investigators (Saw et al., 2014; Zhang and Wu, 2016; Hosseinian et al., 2017; Nejati et al., 2017a,b).

Functionalization of boron nitride (BN) materials has not been exploited extensively despite possessing structural similarities with graphite (Weng et al., 2016). Ding et al. (2017) investigated the combination of phosphorus nanoparticles with boron nitride and graphene as stable anode for sodium-ion batteries (Ding et al., 2017). They reported that αP/c-BN/pGra anodic composition provided an excellent cyclability with stabilized specific capacity of ~1,000 mAhg^−1^ and ~90% retention after 100 cycles at a specific current of 50 mAg^−1^. Similarly, Manthiram (2017) functionalized boron nitride nanosheets and graphene interlayer for a rapid and prolonged duration of lithium-sulfur batteries. Herein, we report for the first time the theoretical investigation into the role of functionalization of boron nitride nanosheet with a conducting polymer, polypyrrole and how it influence the anodic performance of Li, Na, Be, and Mg ion-batteries. The size of lithium-ion has been reported to play a crucial role in the energy storage efficiency of lithium-ion batteries (Manthiram, 2017). Hence the metals were carefully selected to account for the size and charge variations on the storage efficiency of the batteries. A significant improvement in the electronic properties of the BNNS was obtained upon functionalization with PP, reducing the energy gap by 45% of its original value and raising the cell voltages of the metal-ion batteries. This study provides an insight into the role of conducting polymers in improving the energy efficiency of metal-ion batteries and could pave the way for the effective design of highly efficient energy storage materials.

## Computational Methods

Structural optimizations, frontier molecular orbital analysis, and energy predictions were conducted using Grimme's dispersion corrected density functional theory (DFT-D3) (Grimme et al., 2010, 2011), using the hybrid Becke, 3-parameter Lee-Yang-Parr functional (B3LYP) (Becke, 1988; Lee et al., 1988). The starting geometries of the molecules were built on GaussView 5.0 graphical user interface (Dennington et al., 2009), while the simulations were accomplished using Gaussian 09 suite (Frisch et al., 2016). The boron nitride nanosheet, BNNS is made of 63 atoms of B, 63 atoms of N, while the dangling atoms were saturated with hydrogen atoms to reduce the boundary effects (Hosseinian et al., 2017). The Pople's split valence basis set, 6-311G^**^ was adopted for the non-metallic atoms, while for the metals the lanl2dz basis set was chosen. The self-consistent field convergence criterion was set to 1 × 10^−8^ Hartree during the optimizations, whereas the average root means square force on all atoms thresholds was set at 3.0 × 10^−4^ Hartree/Bohr.

Full optimizations were conducted to the minima without enforcing symmetry constraints on the potential energy surface. Reactivity descriptors namely, the energy of the highest occupied molecular orbital, E_HOMO_; energy of the lowest unoccupied molecular orbital, E_LUMO_; the energy gap, E_g_; chemical potential, μ; electronegativity, χ; and the global hardness, η were estimated according to the DFT-Koopman's theorem (Komissarov et al., 2015). The density of state (DOS) plots which represent the nature of electronic structures of the molecules were generated using the Multiwfn code (Lu and Chen, 2012). The adsorption energies of the metal atoms/ions (M/M^n+^) on the pristine BNNS and the polypyrrole functionalized BNNS, PP-BNNS was evaluated using the equation:

(1) $$\begin{array}{r} E_{ad}=\;E_{complex}-\left[E_{sheet/PP}+\;E_{M/M^{n+}}\right] \end{array}$$

Where, *E*_*sheet*/*PP*_ represent the energy of the isolated pristine BNNS or PP-BNNS, *E*_*complex*_ is the energy of the adsorbed metal atoms/ions on the BNNS or PP-BNNS, and $E_{M/M^{n+}}$ represents the energy of the isolated metal atoms/ions.

The changes in energy gap, ΔE_g_ upon adsorption of the metal atoms/ions on the BNNS or PP-BNNS were estimated as an index of the sensitivity of the adsorption as follows:

(2) $$\begin{array}{r} \%E_{g}=\left[\frac{E_{g_{complex}}-\;E_{g_{sheet}}}{E_{g_{sheet}}}\right]\times 100\% \end{array}$$

Where, *E*_*g*_*sheet*__ and *E*_*g*_*complex*__ represents the HOMO-LUMO energy gap of the isolated BNNS or the PP-BNNS, and the energy gap upon adsorption of M/M^n+^, respectively.

Similarly, the initial cell voltages (V_cell_ in volts) were computed using the Nernst equation:

(3) $$\begin{array}{r} V_{cell}=\frac{-\Delta G_{cell}}{zF} \end{array}$$

(4) $$\begin{array}{r} \Delta G_{cell}=\Delta E_{cell}+P\Delta V-T\Delta S \end{array}$$

Where ***z***is the charge of the metal ions (the electrolytic cations) and *F* is the Faraday constant (96500 C/mol or 23.061 kcal/volt.mol). The energy of the overall cell reactions is given by the Gibbs free energy (ΔG_cell_) represented in Equation 4. According to Meng and Arroyo-de Dompablo (2009), the contributions of entropy and the volume effects are negligible (<0.01 V) to the cell voltage (V_cell_), therefore the Gibbs free energy change is equal to the internal energy change of the cell. The ΔE_cell_ represents the change in the energy of adsorption of the M/M^n+^ on BNNS and/or PP-BNNS.

## Results and Discussion

### Structural Properties and Optimization

**Figure 1:**
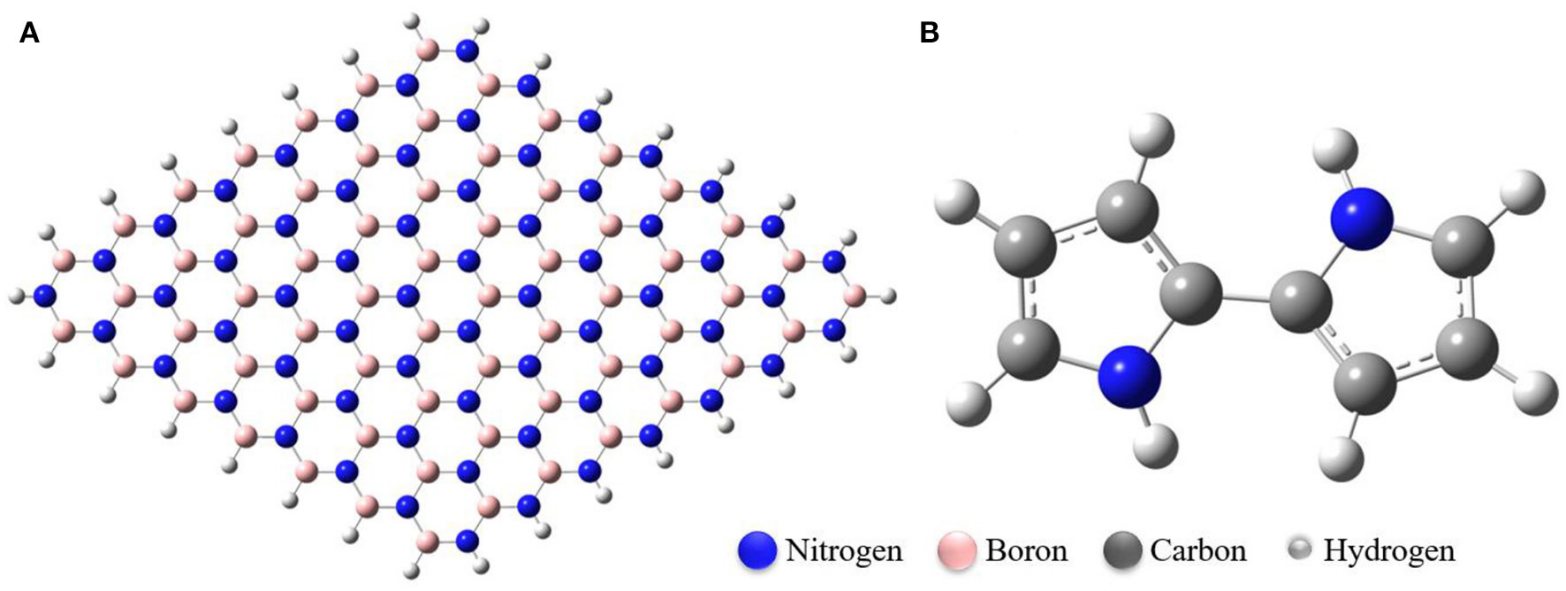
Optimized structures of **(A)** Boron Nitride Nanosheet (BNNS), **(B)** Polypyrrole Molecule (PP). The nanosheet contains 63 boron and nitrogen atoms each.

**Figure 2:**
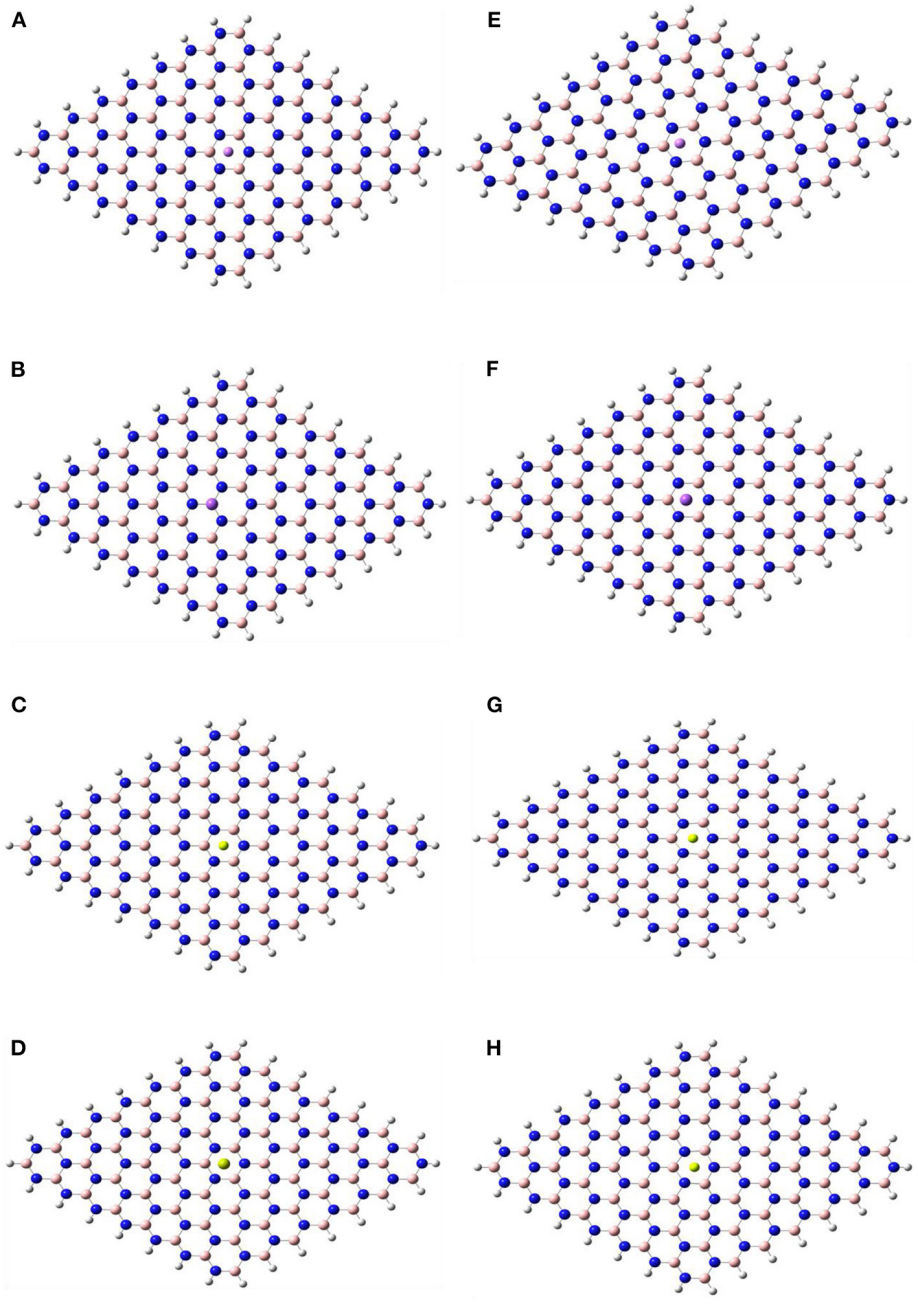
Optimized structures of **(A)** Li@BNNS, **(B)** Na@BNNS, **(C)** Be@BNNS, **(D)** Mg@BNNS, **(E)** Li^+^@BNNS, **(F)** Na^+^@BNNS, **(G)** Be^2+^@BNNS, and **(H)** Mg^2+^@BNNS complexes. See Supplementary Figure 5 in the supplementary for complex structures showing the distance between the M/M^n+^ and the BNNS.

**Figure 3:**
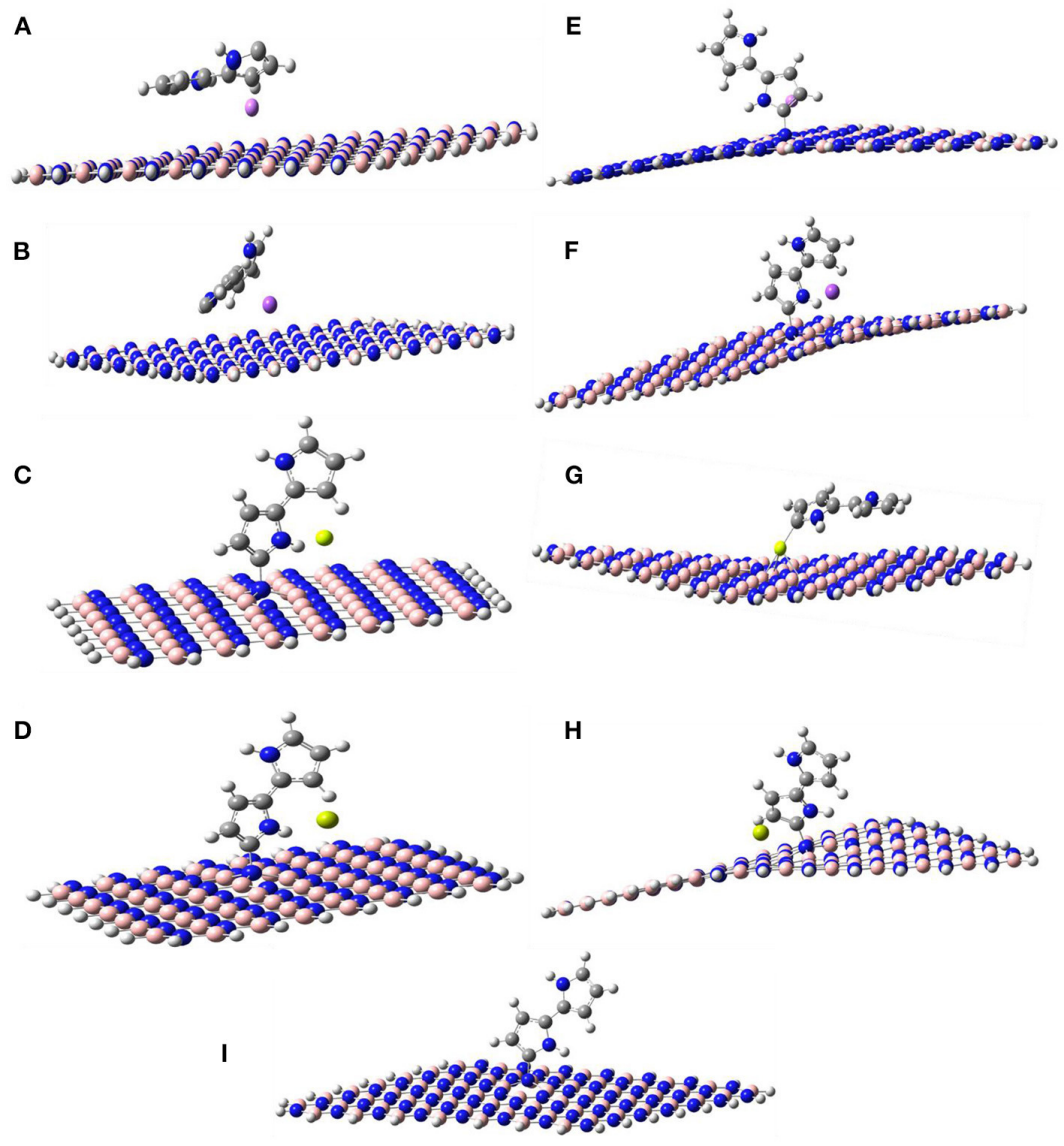
Optimized structures of **(A)** Li@PP-BNNS, **(B)** Na@PP-BNNS, **(C)** Be@PP-BNNS, **(D)** Mg@PP-BNNS, **(E)** Li^+^@PP-BNNS, **(F)** Na^+^@PP-BNNS, **(G)** Be^2+^@PP-BNNS, **(H)** Mg^2+^@PP-BNNS, and **(I)** PP-BNNS complexes. It shows the arrangement of the metal atoms and/or ions, the PP, and the BNNS after optimization. It also shows the distortion in the planarity of the BNNS when it has been functionalized with PP and doped with metal ions **(E–H)**. The degree of the distortion increases as the size of the metal ions increases.

Figures 1, 2 show the optimized structures of the boron nitride nanosheet (BNNS), polypyrrole molecule (PP), and the metals/metal-ions-adsorbed boron nitride nanosheets (M/M^n+^@BNNS). The metals/metal-ions were situated at different locations on the BNNS (on top of the N atoms, B atoms, six-membered ring, and the bridge site of B-N bonds) however, after optimization, it was observed that the metals (Li, Na, Be, and Mg) and the metal-ions (Li^+^, Na^+^, Be^2+^, and Mg^2+^) preferred to relax above the center of the hexagonal ring as shown in Figure 2 and also observed by Hosseinian et al. (2017). The distance between BNNS and M^n+^ in the M^n+^@BNNS complexes is shorter than as in M@BNNS complexes due to stronger interaction (see Supplementary Figure 5 in the supplementary). Similarly, Figure 3 shows that the polypyrrole molecule prefers to bond to the N atom of BNNS after optimization however the atom it was attached to before optimization. For M/M^n+^@PP-BNNS complex, the PP hexagonal ring of attachments is one ring adjacent to the ring suspending the M/M^n+^ at its center. In some cases, such as Be^2+^@PP-BNNS, the PP fragment attaches directly to the Be ion alone while in Li@PP-BNNS and Na@PP-BNNS, the Li and Na atoms were located in-between the horizontally-oriented PP and BNNS structures after optimizations (Figures 3A,B,G, respectively). Furthermore, in Li^+^@PP-BNNS, Be^2+^@PP-BNNS, Na^+^@PP-BNNS, and Mg^2+^@PP-BNNS, the planarity of the BNNS is distorted (Figures 3E–H) with the distortion increasing across the periods of the metal ions. This distortion is believed to occur due to the intense electrostatic interactions between the small energy-gapped PP-BNNS and the empty K and L-shelled metal ions.

**Figure 4:**
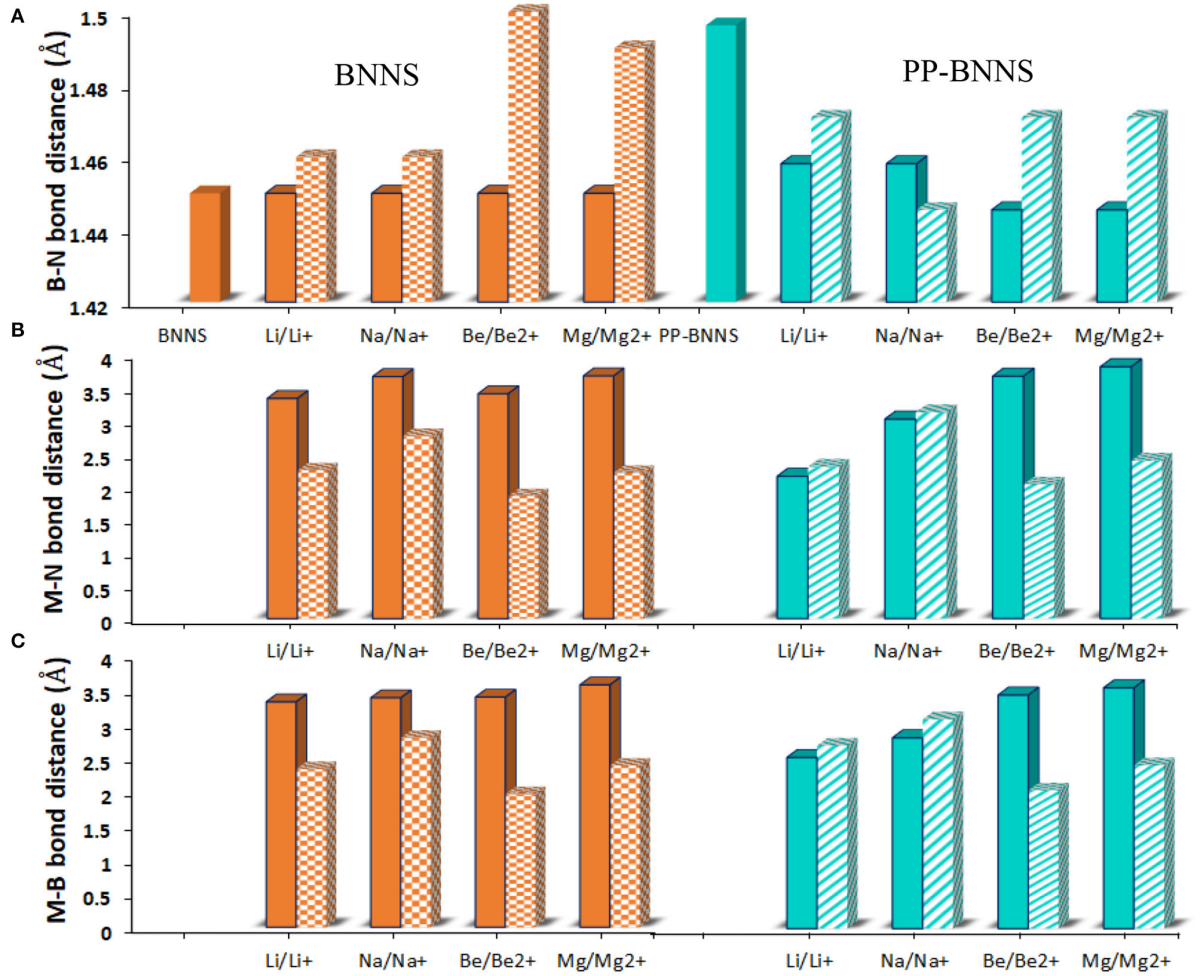
**(A)** the B-N bond distance **(B)** the M-N bond distance and **(C)** the M-B bond distance of BNNS and PP-BNNS, respectively. Where M-N, M^n+^-N, M-B, M^n+^-B, and B-N represent the metal-nitrogen, metal-ion-nitrogen, metals-boron, metal-ion-boron, and boron-nitrogen bonds, respectively. The bond lengths were deduced from the 6-membered ring bearing the PP functional unit. For the PP-BNNS, the B-N bond is deduced from the 6-membered ring bearing the polypyrrole (PP) unit.

As indicated in Figure 4, the B-N bond lengths of BNNS are in the range of 1.44–1.46 Å in agreement with values previously reported (Peyghan et al., 2013; Hosseinian et al., 2017). However, in the presence of polypyrrole fragment, the bond lengths range from 1.42–1.56 Å consequent of the electronic conductivity of the polypyrrole fragment. The B-N bond lengths of the metal-adsorbed BNNS complex (M@BNNS) showed approximately similar values however these values slightly increased for the Be^2+^@BNNS and Mg^2+^@BNNS complexes owing to their smaller size and increased charge. Similarly, the M@PP-BNNS complexes (Li@PP-BNNS, Na@PP-BNNS, Be@PP-BNNS, and Mg@PP-BNNS) showed shorter B-N bond lengths than the ionic counterparts. In the cases of the bond lengths between the metal atoms or metal-ions and B and/or N (M/M^n+^-B and M/M^n+^-N), the values increased down the groups (Li → Na, Li^+^ → Na^+^, Be → Mg, and Be^2+^ → Mg^2+^) and decreases across the period (Li → Be, Li^+^ → Be^2+^, Na → Mg, and Na^+^ → Mg^2+^). This is probably due to the increasing atomic sizes down the groups and the decreasing atomic across the periods. Also, the variations across the periods and down the groups may also likely contribute to these changes in bond lengths among these complexes which suggests that charge and size effects of these metallic and/or ionic dopants have effects on the structural properties of these complexes.

### Effect of Polypyrrole Functionalization

**Figure 5:**
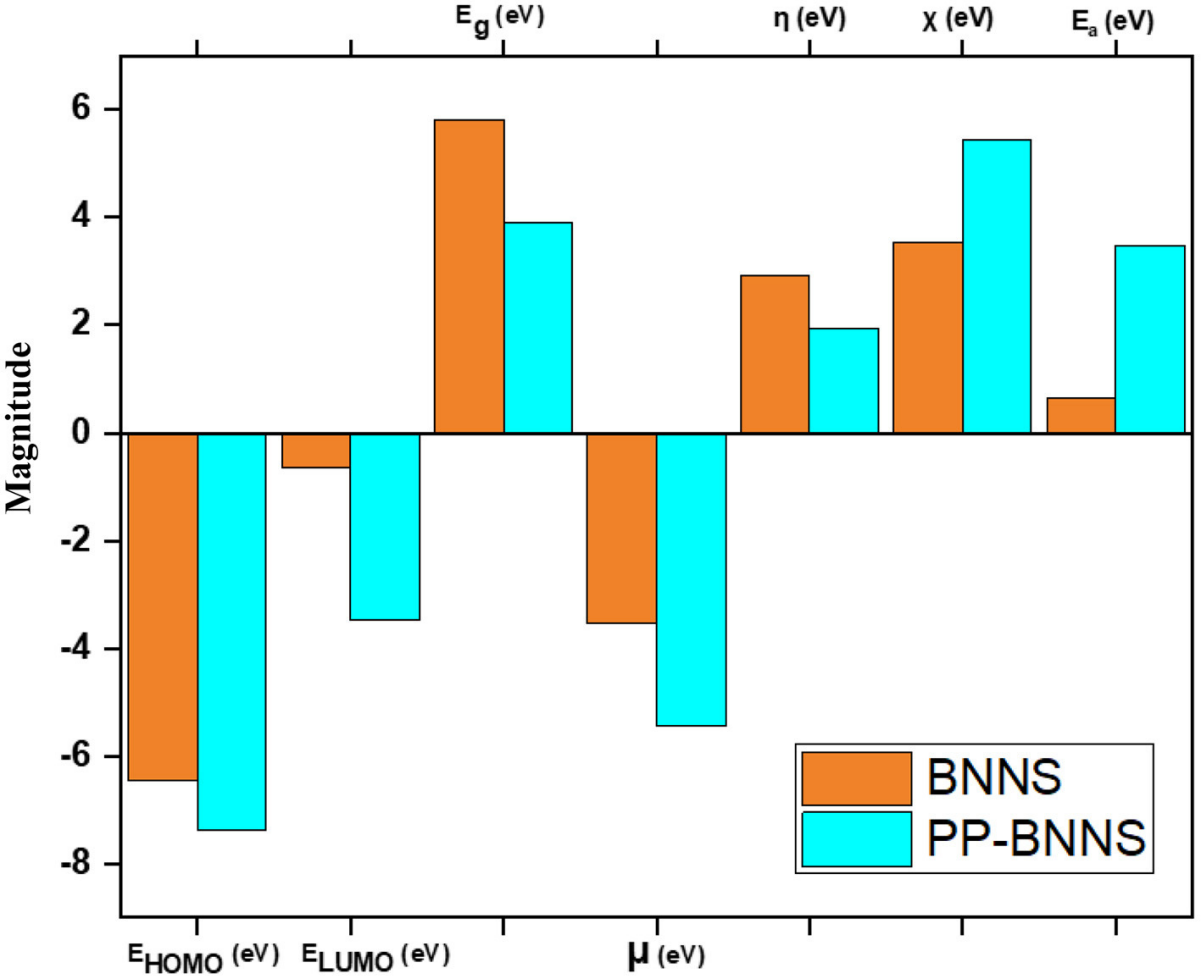
Electronic properties of BNNS and PP-BNNS. It shows the LUMO-HOMO energy levels, energy gap (E_g_), chemical potential (μ), global hardness (η), electronegativity (χ), and the electron affinity of BNNS and PP-BNNS.

**Figure 6:**
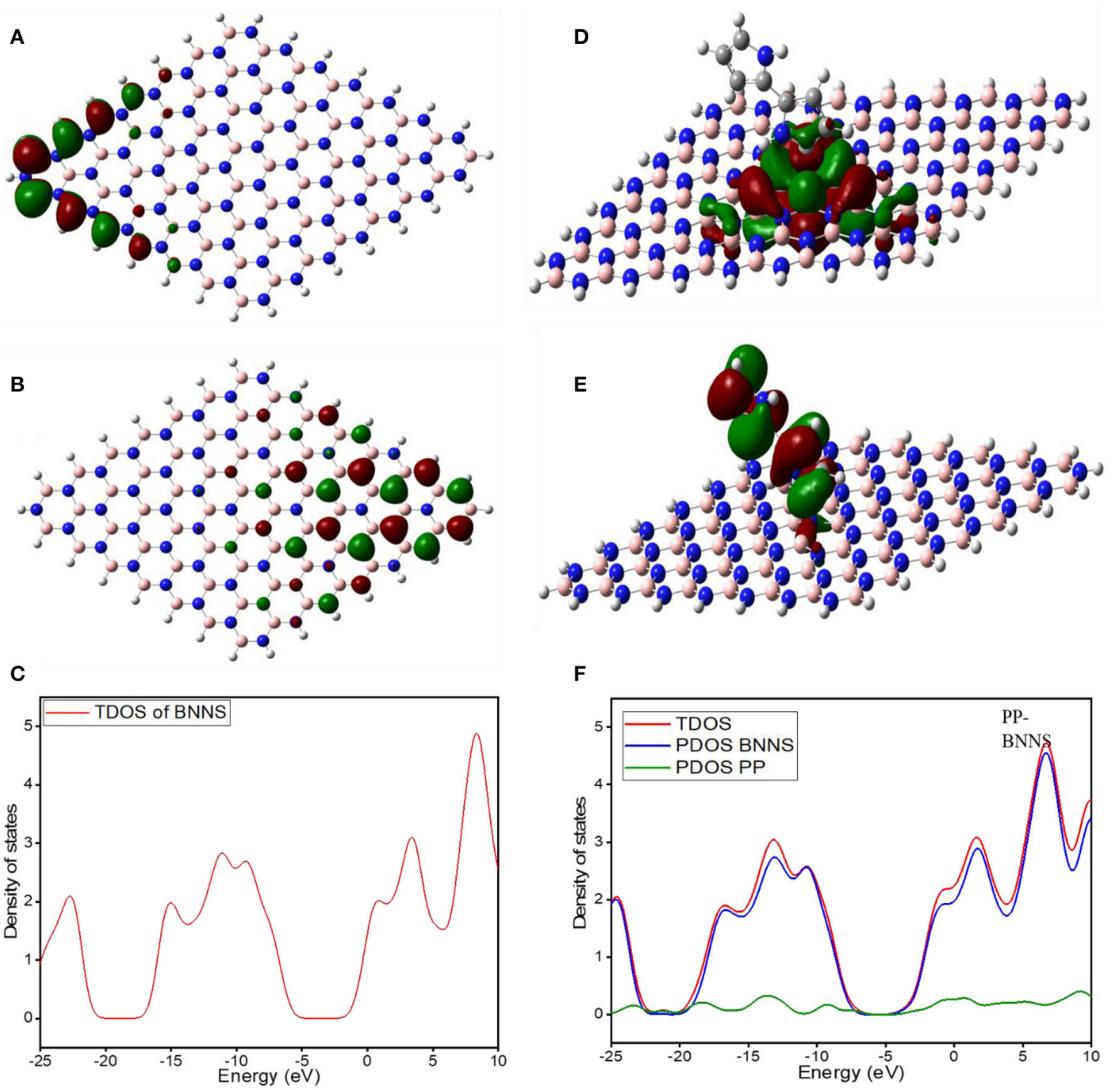
**(A–C)** show the molecular orbital LUMO, HOMO, and the DOS of BNNS, while **(D-F)** show the molecular orbital LUMO, HOMO, and the DOS of PP-BNNS, respectively. The HOMO and LUMO of BNNS are located largely around the B and N atoms. Similarly, the HOMO of PP-BNNS is largely located around the PP fragment while the LUMO is located around the BN rings surrounding the PP fragment.

Figure 5 shows the electronic properties of the pristine BNNS and the polypyrrole functionalized boron nitride nanosheet (PP-BNNS). The energy gap (E_g_) of BNNS obtained is comparable with the experimental band gap value of ~5.50 eV (Song et al., 2010; Hosseinian et al., 2017). However, the presence of PP decreased the energy gap of the BNNS by over 45%, thus suggesting an increase in the reactivity of the BNNS. It can also be predicted that the PP-BNNS will exhibit superior electronic interactions with an adsorbate due to its smaller energy gap (Abdulazeez et al., 2019). As shown in Figure 6, the HOMO and LUMO of BNNS are located largely around the B and N atoms (Peyghan et al., 2013). Similarly, the HOMO of PP-BNNS is largely located around the PP fragment while the LUMO is located around the BN rings surrounding the PP fragment. This may explain the reason for the reduction in the energy gap of BNNS after functionalization with PP as shown in Figure 5.

Furthermore, the effect of PP on the BNNS can be deduced from the higher chemical potential (μ, eV), lower global hardness (η, eV), higher electronegativity (χ, eV) and higher electron affinity (Ea, eV) for the PP-BNNS complex. The global hardness, η which measures the resistance of molecules to electron density distortion is an essential electronic property that reveals the ability of the nanosheets to donate their non-bonding electrons during interaction (Abdulazeez et al., 2019). According to the hard-soft acid-base (HSAB) theory, molecules with high hardness exhibit poor reactivity as a result of their lower tendency to donate electrons. However, soft molecules (lower η values) have better electron-donating tendencies and are regarded as effective in the adsorption process (Chattaraj et al., 2002; Wang et al., 2007).

Figures 6C,F shows the curves of the total density of state (TDOS) for BNNS, and the total and partial density of states for PP-BNNS, BNNS, and PP, respectively. A direct view on adsorbate surface interaction is possible by looking at the DOS (Hussain et al., 2020). This quantity is directly accessible in DFT calculations. The total density of states (TDOS) comprises all the electrons within the system. To reveal the electronic orbital changes of involved orbitals of the adsorbate and the substrate during the adsorption process, DOS decomposes into its building components which can be achieved by calculating the partial density of states (PDOS) (Shou et al., 2019). One of the most important applications of DOS plots is the pictorial representation of molecular orbitals and their contribution to adsorption. The orbitals hybridization of BNNS and PP confirmed the adsorption of PP on the BNNS sheet. The change of position of frontier molecular orbitals was observed and the gap between them is decreased upon the adsorption of PP on BNNS. The positive values of DOS present bonding interaction, negative values mean anti-bonding interaction, and zero values indicate non-bonding interactions.

### Metals/Metal-Ions Adsorption on BNNS and PP-BNNS

**Figure 7:**
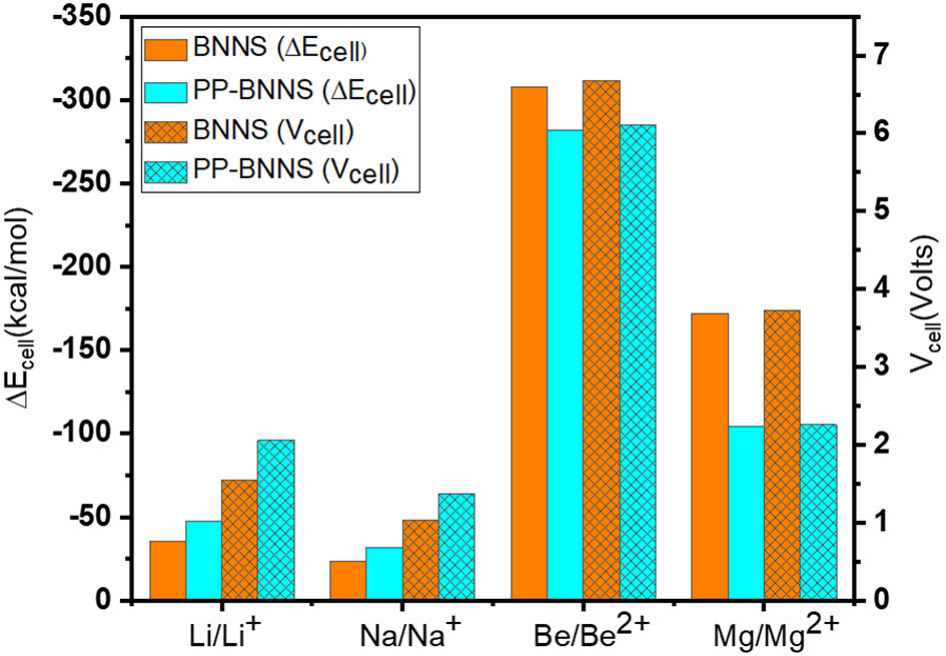
Energy changes of the cell (ΔE_cell_) and the cell voltages of M/M^n+^@BNNS and M/M^n+^@PP-BNNS.

To study the adsorption natures of BNNS and PP-BNNS, the metal atoms and ions (M and M^n+^) were oriented as discussed in section Structural Properties and Optimization. Figure 7 gives the ΔE_cell_ of BNNS (orange bar) and PP-BNNS (cyan bar) resulting from the energies of adsorption of M/M^n+^ on BNNS and PP-BNNS, and the generated voltages of the cells. The adsorption energies of the metal ions (Li^+^, Na^+^, Be^2+^, and Mg^2+^) on BNNS are remarkably more negative than their metal atoms (see Supplementary Table 1). However, for the adsorption on PP-BNNS, Li, Na, Be^2+^, and Mg^2+^ have more negative adsorption energies than their respective atoms and/or ion species. According to charge analysis, there is a greater charge interaction between the three N atoms and the metal ions than with metal atoms. These may likely be responsible for the weaker interaction of the metal atoms with the BN nanosheet than the metal ions. The adsorption trend with PP-BNNS may be attributed to the electronic contributions of the PP fragment and the orientation of the M/M^n+^@PP-BNNS complexes as described in Section Structural Properties and Optimization.

Supplementary Table 1 also shows the effects of the metal atoms and ions on the electronic properties of BNNS and PP-BNNS complexes. The adsorption of the metal ions (Li^+^, Na^+^, Be^2+^, and Mg^2+^) on the BNNS and PP-BNNS generally stabilized the HOMO and LUMO energies and resulted in largely decreased energy gaps (E_g_, eV) by over 85% for Be^2+^ and Mg^2+^, and 40% for Li^+^ and Na^+^, except for Li^+^@PP-BNNS and Na^+^@PP-BNNS with contrary E_g_ (eV) values. Similarly, the metal atoms adsorption on the BNNS and PP-BNNS have a destabilization effect on the complexes supposedly due to the unpaired electrons of the metal atoms. However, the influence of group one metal atoms (Li and Na) on the E_g_ of BNNS complexes is less significantly destabilizing compared to those of their ions, while the group two atoms (Be and Mg) have a much more destabilization influence on the E_g_ of BNNS than their respective ions. The trend is uniform for the PP-BNNS complexes with the metal ions having much more stabilization influence on the E_g_ of the complexes than their respective ions with destabilization influence.

To further investigate the effect of adsorption of metal atoms or metal ions on the electronic structure of BNNS and PP-BNNS complexes, the total density of states (TDOS) and the partial density of states of the complexes were evaluated as shown in Supplementary Figures 1, 2. The position of HOMO orbitals has been slightly altered upon the adsorption of metal atoms or metal ions on the BNNS sheets. The adsorption of metal atoms or metal ions on the PP-BNNS complex, modified the electronic properties of the complex, and the position of the bonding and anti-bonding molecular orbitals were changed, and the energy gaps were narrowed as seen in Supplementary Figure 2.

### Application of BNNS and PP-BNNS to Ion-Batteries

The BNNS and PP-BNNS are considered as the anodes for the ion-batteries where the reactions taking place at the cathodic and anodic half cells are as shown in Equations 5, 6, and also similar to previous literature reports (Datta et al., 2014; Bagheri, 2016; Gao et al., 2016). The overall reaction of the cells is as shown in Equation 7.

(5) $$\begin{array}{l} \text{Anode}:M@sheet↔M^{\text{n}+}@sheet+ne^{-} \end{array}$$

(5a) $$\begin{array}{r} \text{Discharge}:M@sheet\to M^{\text{n}+}@sheet+ne^{-} \end{array}$$

(5b) $$\begin{array}{r} Charge:M^{\text{n}+}@sheet+ne^{-}\to M@sheet \end{array}$$

(6) $$\begin{array}{r} \text{Cathode}:M^{\text{n}+}+ne^{-}↔M \end{array}$$

(7) $$\begin{array}{r} Overall\;cell\;reaction:M^{\text{n}+}+M@sheet\\ ↔M^{\text{n}+}@sheet+M+\Delta G_{\text{cell}} \end{array}$$

Where M is the metal atoms, M^n+^ is the metal ions and “sheet” is the BNNS and/or PP-BNNS. As shown in Supplementary Table 1, the adsorption energies of the metal ions (M^n+^) on BNNS and PP-BNNS, are more electronegative than the metal atoms (M) except for Li^+^@PP-BNNS and Na^+^@PP-BNNS complexes with reverse outcomes (probably due to their increased energy gaps). The complexes with the more negative energy of adsorption gave more favorable and stronger interactions with their respective nanosheets. The change in internal or adsorption energies (ΔE_cell_, Kcal/mol) of the cells negatively decreases down the group of the metals and increases across their periods irrespective of the nanosheet. This may suggest the effect of increased charge and size reduction contributions of the metal ions toward favorable and stronger interactions with the nanosheets and the subsequent cell voltages.

Furthermore, the cell voltages are shown in Figure 7 where it is obvious that the V_cell_ values are affected by the size and charge of the ions and the presence of the conducting polymer (Polypyrrole, PP). The V_cell_ for Li-BNNS complex (1.55 V) is comparable with established voltages; 1.45 V (Gao et al., 2016), 1.46 V (Hosseinian et al., 2017), and 1.70 V (Bagheri, 2016). As the size of the metal ions increases, the established voltages decreases while as the charge of the metal ions increases, the voltages increase. This is another indication that the charges on the metal ions and their sizes likely affect the voltage of the cells and their subsequent use as anodic materials in ion-battery systems. The Be-nanosheet has the highest cell voltage for the two considered nanosheets (BNNS and PP-BNNS) followed by Mg-nanosheet. This is also reflective of their longer bond lengths and the ease of mobility of their ions during charging and discharging when compared to the rest of the metals. Generally, functionalization of the BNNS with PP, improved the cell voltages of Li- and Na-sheets, suggesting that conducting polymer materials may have a tremendous contribution toward improving the energy storage capacity of LIBs and SIBs if further investigated. It is a fact that larger cell voltage (V_cell_) contributes toward storage performances of batteries and the rise in their discharge rates. Therefore, the BNNS and PP-BNNS complexes may be suitable for applications in anodic ion-batteries including lithium-ion batteries, sodium-ion batteries, beryllium-ion batteries, and magnesium-ion batteries.

## Conclusion

We have investigated the effect of functionalization of boron nitride nanosheet, BNNS with conducting polymer, polypyrrole using first-principles DFT calculations, and evaluates its potential as anode material for Li, Na, Be, and Mg-ion batteries. The electronic structural analysis shows that the functionalization of the BNNS with PP significantly reduced the energy gap of BNNS by over 45% (from 5.809 to 3.901 eV) and improved other electronic properties of BNNS such as global hardness, chemical potential, and cell reaction kinetics. A significant improvement in the cell voltage, ΔE_cell_ was estimated with PP functionalization specifically for Li-ion (from 1.55 to 2.06 V) and Na ion (from 1.03 to 1.37 V), the trend of which revealed the influence of the size and the charge on the metal ions in promoting the energy efficiency of the batteries. Our results demonstrate the role of conducting polymers in enhancing the energy performance of Li, Na, Be, and Mg-ion batteries and could pave the way for the effective design of highly efficient energy storage materials.

## Data Availability Statement

The raw data supporting the conclusions of this article will be made available by the authors, without undue reservation.

## Author Contributions

CN, IA, and MH carried out the DFT calculations and contributed to writing the manuscript draft and interpreted the results. AJ and QP wrote parts of the draft and interpreted the results. AA-S conceived the idea and interpreted the results. All authors read and approved the final draft.

## Conflict of Interest

The authors declare that the research was conducted in the absence of any commercial or financial relationships that could be construed as a potential conflict of interest.
