# Supplementary material for: Impact of Polypyrrole Functionalization on the Anodic Performance of Boron Nitride Nanosheets: Insights From First-Principles Calculations
Source: Front Chem. 2021 Apr 28;9:670833. doi: 10.3389/fchem.2021.670833 (PMC8113678; doi:10.3389/fchem.2021.670833)
Supplement: Supplementary file 1 [file Data_Sheet_1.pdf]

Supporting Information

Impact of polypyrrole functionalization on the anodic performance of boron nitride nanosheets: insights from first-principles calculations

Chidera C. Nnadiekwe[a], Ismail Abdulazeez[a], Muhammad Haroon[a], Qing Peng[b,c], Almaz Jalilov[a], Abdulaziz Al-Saadi[a,*]

[a]Chemistry Department, King Fahd University of Petroleum and Minerals, Dhahran 31261, Saudi Arabia

[b]Physics Department, King Fahd University of Petroleum and Minerals, Dhahran 31261, Saudi Arabia

[c]K.A CARE Energy Research & Innovations Center at Dhahran, Dhahran 31261, Saudi Arabia

***** *Corresponding author: Dr. Abdulaziz Al-Saadi (asaadi@kfupm.edu.sa)*

Contents

1. **Density of states of the metal atoms/ions adsorbed onto BNNS.**
2. **Density of states of metal atoms/ions adsorbed onto PP-BNNS.**
3. **HOMO-LUMO maps of the metal atoms/ions on BNNS.**
4. **HOMO-LUMO maps of the metal atoms/ions on PP-BNNS.**
5. **Optimized structural cross-sections of the metal atoms/ions onto BNNS.**
6. **Adsorption energies of the metal atoms/ions on BNNS and PP-BNNS.**

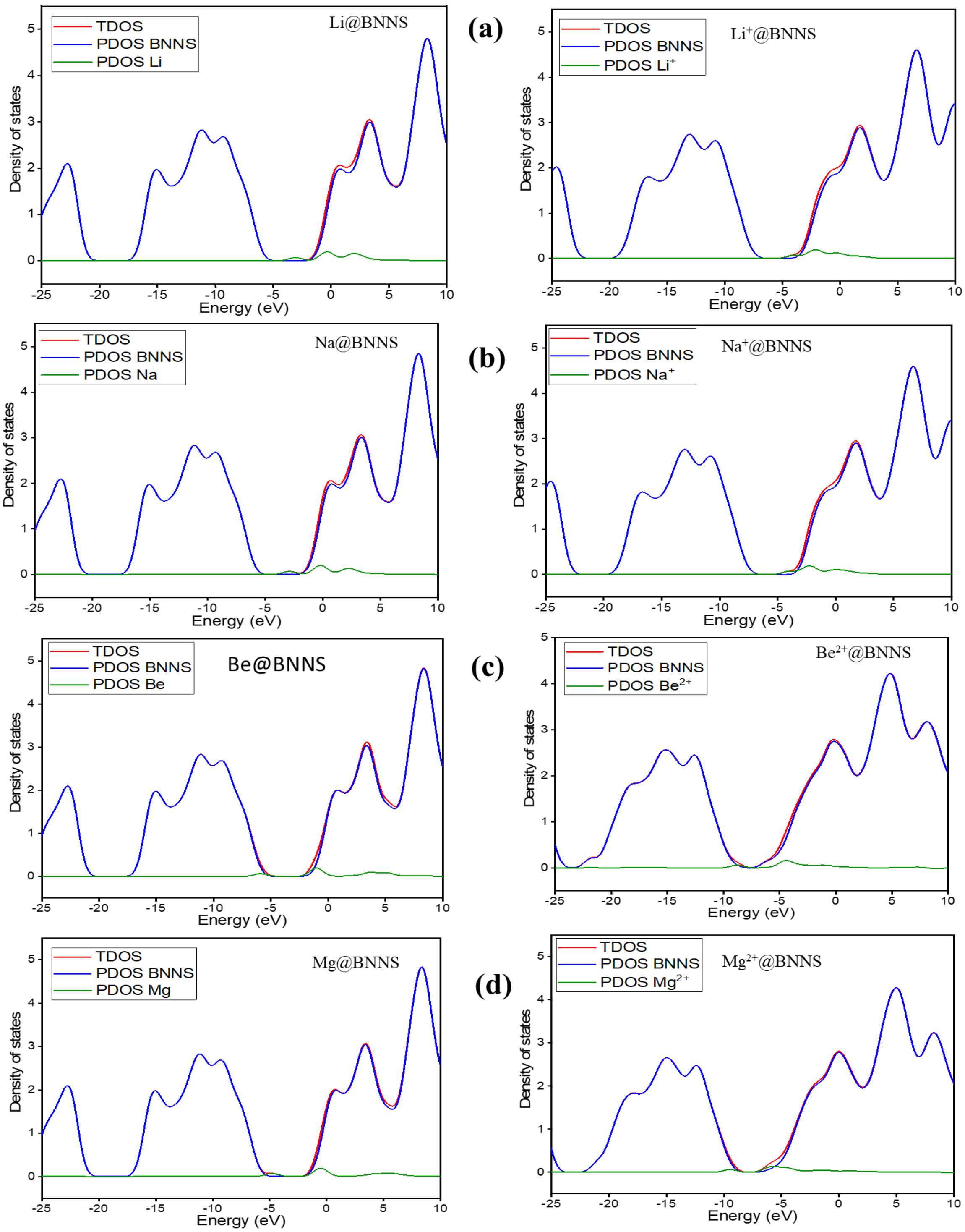

Figure S1: Density of States of the metal atoms and ions adsorbed onto the BNNS. It shows the total and partial state of the complexes and/or their components.

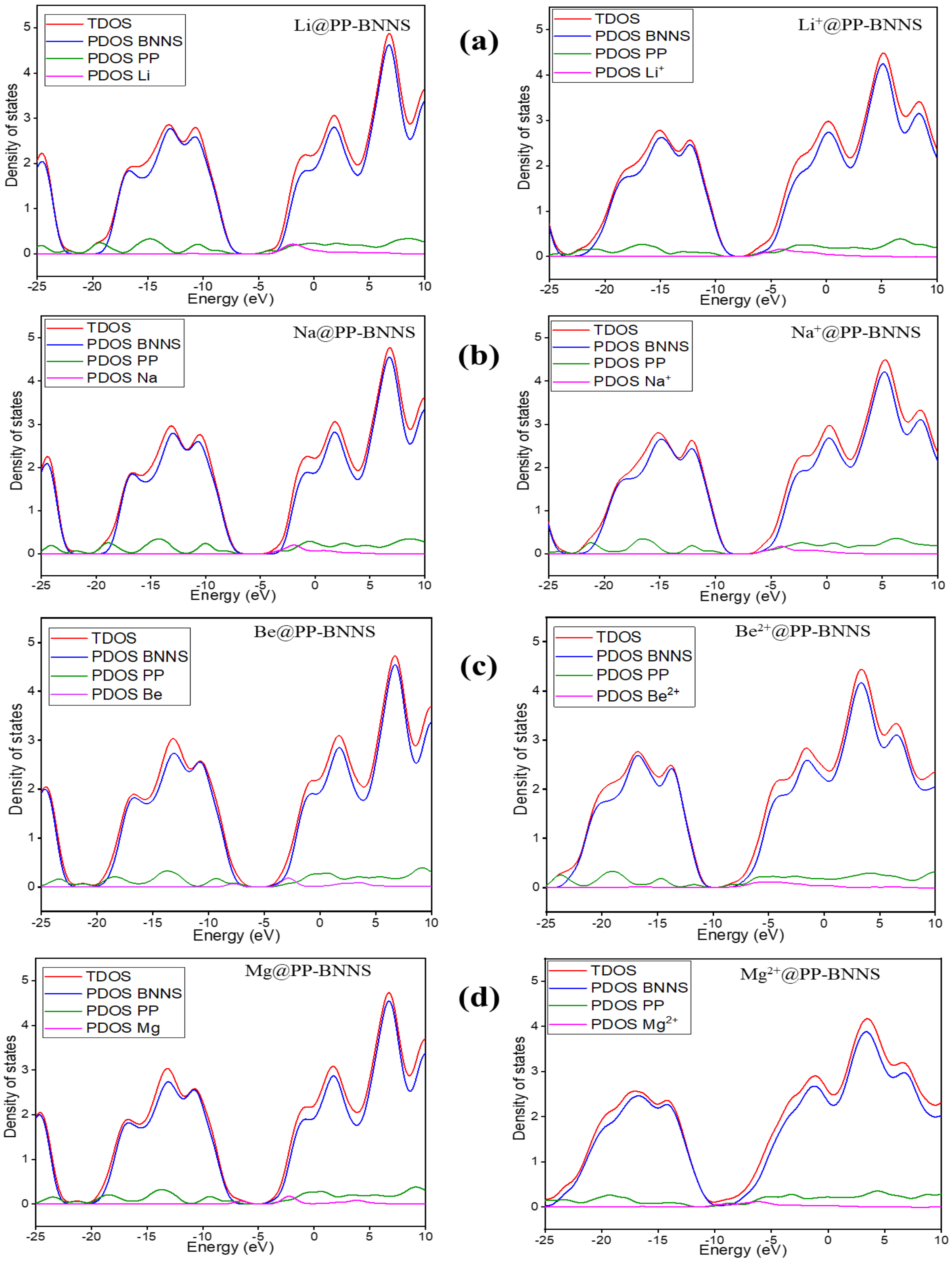

Figure S2: Density of States of the metal atoms and ions adsorbed onto the PP-BNNS. It shows the total and partial densities of the state of the complexes and/or their components.

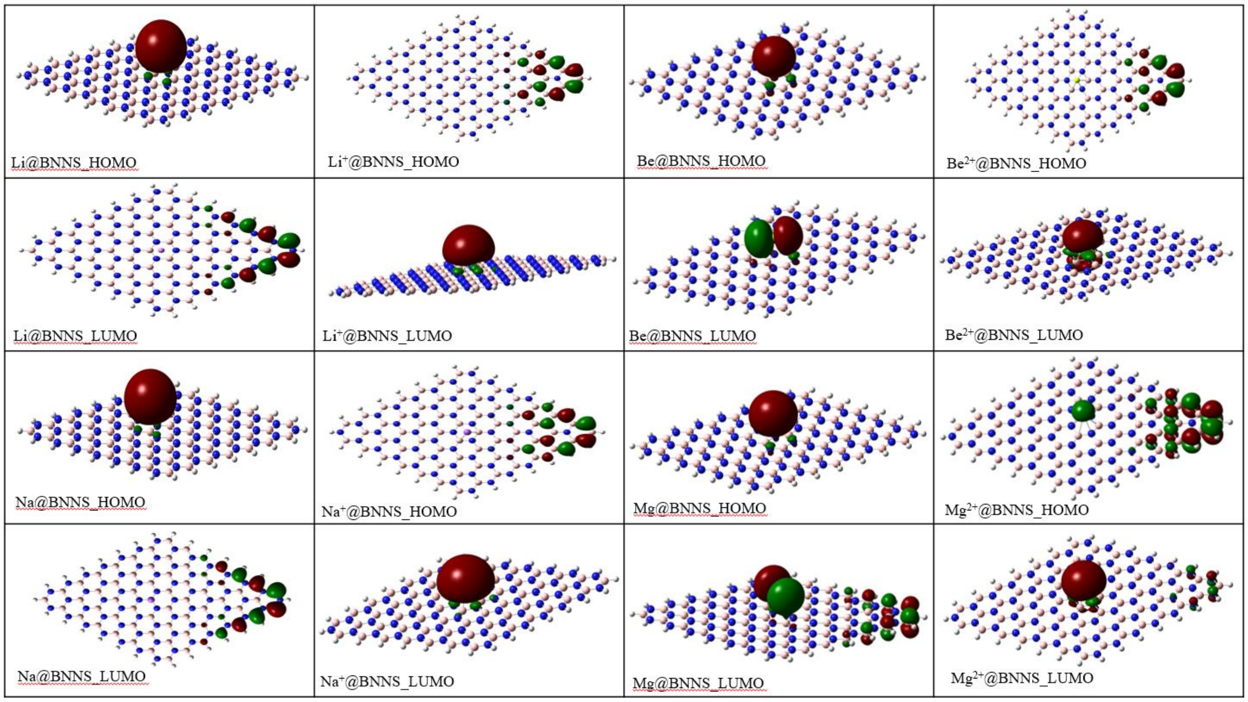

Figure S3: The HOMO and LUMO maps of the metal atoms and/or ions adsorbed onto the BNNS (M/M^{n+}@BNNS).

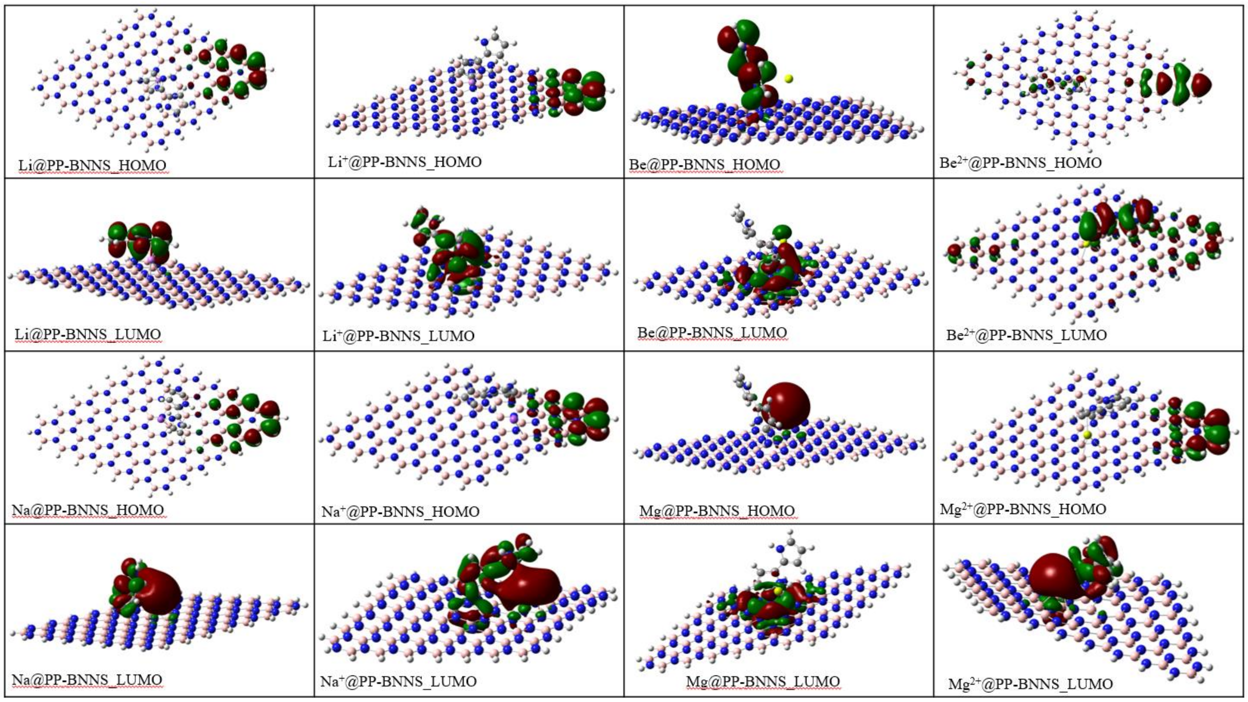

Figure S4: The HOMO and LUMO maps of the metal atoms and/or ions adsorbed onto the PP-BNNS (M/M^{n+}@PP-BNNS).

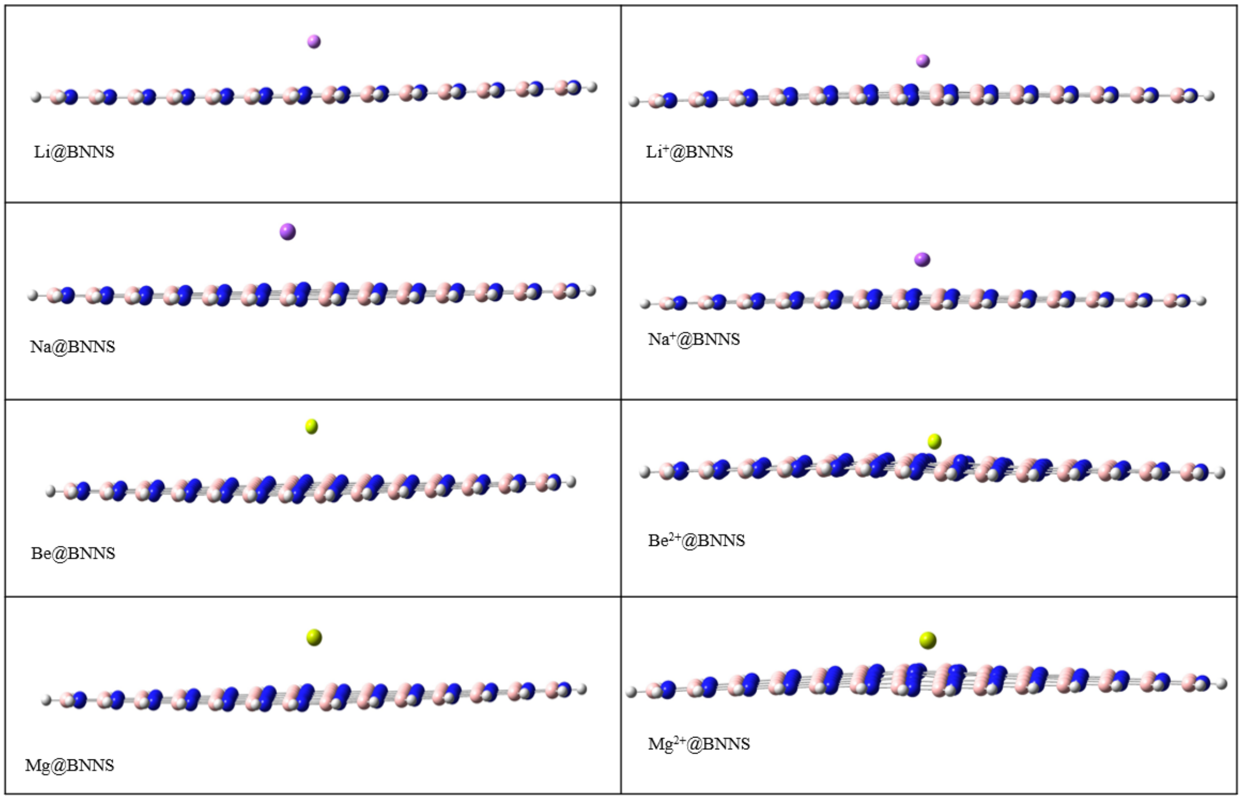

Figure S5: Optimized structural cross-section of (a) Li@BNNS, (b) Na@BNNS, (c) Be@BNNS, (d) Mg@BNNS, (e) Li^{+}@BNNS, (f) Na^{+}@BNNS, (g) Be^{2+}@BNNS, and (h) Mg^{2+}@BNNS complexes showing the distance between the M/M^{n+} and the BNNS. It can be seen that the distance is shorter for M^{n+}@BNNS than M@BNNS due to strong adsorption.

Table S1: Adsorption energies of metal atoms and ions on BNNS and PP-BNNS complexes

	E_{HOMO} (eV)	E_{LUMO} (eV)	E_g (eV)	%ΔE_g	E_{ad} (kcal/mol)
BNNS	-6.449	-0.639	5.809	-	-
PP-BNNS	-7.380	-3.479	3.901	-32.846	-542.71
Li^+@BNNS	-7.979	-4.065	3.914	-32.622	-41.12
Li@BNNS	-3.070	-0.662	2.409	-58.530	-5.45
Na^+@BNNS	-7.953	-3.994	3.959	-31.847	-25.08
Na@BNNS	-2.906	-0.655	2.251	-61.250	-1.82
Be^{2+}@BNNS	-9.465	-8.815	0.650	-88.810	-308.73
Be@BNNS	-5.877	-1.109	4.768	-17.920	-0.79
Mg^{2+}@BNNS	-9.695	-9.456	0.239	-95.886	-173.83
Mg@BNNS	-4.801	-0.654	4.147	-28.611	-1.57
Li^+@PP-BNNS	-9.500	-6.318	3.182	-18.431	-1.55
Li@PP-BNNS	-8.014	-3.975	4.038	3.512	-49.17
Na^+@PP-BNNS	-9.787	-5.548	4.240	8.690	-8.57
Na@PP-BNNS	-8.037	-3.444	4.594	17.765	-40.24
Be^{2+}@PP-BNNS	-11.761	-11.573	0.188	-95.181	-282.29
Be@PP-BNNS	-7.446	-3.539	3.907	0.154	-0.210
Mg^{2+}@PP-BNNS	-10.886	-9.685	1.201	-69.213	-106.99
Mg@PP-BNNS	-6.592	-3.518	3.074	-21.120	-2.69
